# Reusable and single-use endoscopes: infection prevention and sustainability considerations – a narrative review

**DOI:** 10.3205/dgkh000674

**Published:** 2026-09-10

**Authors:** Christoph Lübbert, Nico T. Mutters, Simone Scheithauer, Heike von Baum, Alexander Meining, Andreas Rieß, Marc Thanheiser, Franziska Lexow, Jana Maidhof, Axel Kramer

**Affiliations:** 1 Division of Infectious Diseases and Tropical Medicine, Department of Medicine I, Leipzig University Medical Center, Leipzig, Germany; 2 Department of Infectious Diseases and Tropical Medicine, Hospital St. Georg gGmbH, Leipzig, Germany; 3 Institute for Hygiene and Public Health, University Hospital Bonn, Bonn, Germany; 4 Department of Infection Control and Infectious Diseases, University Medical Center Göttingen (UMG), Georg-August University Göttingen, Göttingen, Germany; 5 Institute of Medical Microbiology and Hygiene, University Hospital of Ulm, Ulm, Germany; 6 Interventional and Experimental Endoscopy (InExEn), Department of Internal Medicine 2, University Hospital Würzburg, Würzburg, Germany; 7 Josefs-Gesellschaft gAG, Cologne, Germany; 8 Department for Infectious Diseases, Unit 14: Hospital Hygiene, Infection Prevention and Control, Robert Koch Institute, Berlin, Germany; 9 Institute of Hygiene and Environmental Medicine, University Medicine Greifswald, Greifswald, Germany

**Keywords:** economical break-even point, narrative review, endoscopes, bronchoscopes, gastroscopes, duodenoscopes, cholangioscopes, ureteroscopes, cystoscopes, infection prevention, reusable, single-use, sustainability assessment

## Abstract

Despite the greater effort required for reprocessing, reusable endoscopes may be environmentally more beneficial than disposable products, particularly when used frequently and with optimized reprocessing procedures. Single-use endoscopes generate more waste and require more resources per usage but may offer advantages in certain situations with regard to infection prevention and logistical simplicity. It is crucial to carefully weigh environmental considerations, patient safety, practical feasibility, and economic factors. For both systems, sustainability considerations must be taken into account in addition to their targeted, indication-specific use. Facility-specific decisions regarding the use of reusable versus single-use endoscopes require knowledge of the patients being treated, the scope of care, care structures, and procedure-specific risks.

## Abstract (de)

Mehrwegendoskope können trotz ihres höheren Aufbereitungsaufwands ökologisch vorteilhafter sein als Einwegendoskope, insbesondere bei hoher Nutzungsfrequenz und optimierten Aufbereitungsprozessen. Einwegendoskope verursachen mehr Abfall und höheren Ressourcenverbrauch pro Anwendung, können in bestimmten Situationen aber Vorteile hinsichtlich Infektionsprävention und logistischer Einfachheit bieten. Entscheidend ist die differenzierte Abwägung zwischen Patientensicherheit, Umweltverträglichkeit, praktischer Umsetzbarkeit und ökonomischen Aspekten. Für beide Systeme sind neben dem gezielten, indikationsbezogenen Einsatz Nachhaltigkeitsaspekte zu berücksichtigen. Die einrichtungsinternen Abwägungen zur Verwendung von Mehrweg- vs. Einwegendoskopen setzen die Kenntnis der zu versorgenden Patientinnen und Patienten, des Versorgungsumfangs, der standortspezifischen Versorgungsstrukturen und der eingriffsspezifischen Risiken voraus.

## Preamble

The Commission for Infection Prevention and Hygiene in Healthcare and Nursing (KRINKO) has made it its mission to take aspects of sustainability into account when developing recommendations, and accordingly, relevant research is being conducted. This narrative review was prepared as part of the work of a KRINKO working group, but does not constitute an official recommendation or statement by the commission.

## 1 Introduction

In 1987, the United Nations World Commission on Environment and Development defined sustainable development as *„…development that meets the needs of the present without compromising the ability of future generations to meet their own needs.”* [1]. The health sector, in particular, plays an important role in tackling health problems caused by climate change, as it itself contributes to greenhouse gas emissions both directly and indirectly [2]. The World Health Organization (WHO) therefore sees great potential in the health sector to contribute to climate protection through more sustainable structures and processes [3]. This applies in particular to infection prevention measures, as these are essential but consume significant resources, for example the manufacture and packaging of products, the use of energy and water, the consumption involved in the reprocessing of reusable products, the generation of waste and wastewater, and the release of ecotoxicologically relevant antimicrobial agents into the environment. For this reason, every infection-prevention measure should be subjected to a careful risk-benefit assessment, with justification for its use. Where measures have comparable effectiveness, the one with the lower carbon footprint should be selected [4], [5].

**The aim of this narrative review is to analyze the potential for optimizing the use of reusable and single-use endoscopes. The challenge lies in striking the best possible balance between providing optimal medical care for patients, including the protection of staff, and acting in a sustainable manner.**

Endoscopic diagnosis and treatment are firmly established in many inpatient and outpatient medical facilities. In Germany, the Commission for Infection Prevention and Hygiene in Healthcare and Nursing (KRINKO) has addressed the infection control and prevention aspects of endoscopy in various guidelines (e.g., [6], [7]). In this context, the focus of prevention lies on validated reprocessing. KRINKO states in general terms that the reprocessing of medical devices should be critically assessed in terms of cost-effectiveness and environmental considerations [6].

Pursuant to section 8 (1) of the Ordinance on Operators of Medical Devices (*MPBetreibV*), the reprocessing of medical devices must be carried out using suitable, validated procedures, taking into account the manufacturer’s instructions, so as not to endanger the safety and health of patients, users, and third parties [8]. This applies to every reusable endoscope and its reusable components (e.g., distal attachment cap, valves) [7]. Adherence to an appropriate reprocessing procedure places high demands on human and material resources (for instance, chemicals, water, energy), including the necessary expertise and quality assurance. At first glance, the use of reusable instead of single-use products appears to be a simple way of improving sustainability. However, it must be borne in mind that the life-cycle assessment is often difficult to estimate or calculate, because when reusable products are used, factors such as the consumption of water, energy and chemicals, as well as any transport distances involved, must be considered for their proper reprocessing. Ultimately, the service life of the reusable product, including its individual components, is also factored into the life-cycle assessment. On the other hand, for single-use products, both the costs of manufacturing (e.g., raw material consumption, energy) and the volume of waste generated must be taken into account. Finally, practical considerations regarding the use of the medical device can influence the choice. In the case of reusable and single-use endoscopes, this relates to the size of the endoscopy department or the number of endoscopes required for intraoperative use or for patients receiving intensive care, among other factors. Furthermore, the use of reusable or single-use endoscopes depends not only on the clinical indication but also to a considerable extent on the number of endoscopies performed in a year.

To ensure this narrative review is as clear as possible, it is not possible to go into detail about the wide variety of endoscope types. The content therefore focuses primarily on flexible gastrointestinal endoscopes and bronchoscopes, as these are the most commonly used types of endoscopes, and cannot be readily applied to other types of endoscopes. The main criteria for determining whether to use reusable or single-use endoscopes are the quality of care and patient safety. In this context, the focus is primarily on infection prevention criteria to support decision-making. In addition to the direct product characteristics and process parameters relating to reprocessing, the infection prevention assessment must also take into account further structural parameters such as the size and layout of the facility, the number of patients to be treated, and the associated treatment particulars. Furthermore, the assessment of whether to opt for reusable or single-use endoscopes has to take into account advantages for diagnosis and treatment, technical complexity, and design aspects related to product handling, as well as economic considerations.

## 2 Reasons for comparing reusable and single-use endoscopes from an infection prevention perspective

The reason for this comparison is the increased availability of single-use endoscopes against the backdrop of endoscope-associated pathogen transmission. The development of single-use endoscopes is generally justified by the postulated argument that they offer greater protection against infection. In earlier studies, the incidence of endoscope-associated infection was estimated at 1:1.8 million [9], [10], although the overall infection rate during duodenoscopy with therapeutic endoscopic retrograde cholangiography (ERC) or endoscopic retrograde cholangiopancreatography (ERCP) can rise to as high as 2–4% when endogenous infections are included [11].

Following duodenoscopy with ERC/ERCP, the risk of endoscope-associated, contamination-related infection is estimated to be at least 0.01%. Therefore, associated clusters and outbreaks are possible; for example, between 2008 and 2018, three outbreaks caused by duodenoscopes were reported in the Netherlands [12]. A systematic review covering the period 1990–2004 reported one outbreak worldwide linked to arthroscopes, three linked to cystoscopes, seven linked to duodenoscopes used in ERCP, ten linked to endoscopes for the upper gastrointestinal tract, 12 linked to endoscopes for the lower gastrointestinal tract, and 35 linked to bronchoscopes [13]. Since then, the quality of reprocessing has improved worldwide thanks to quality assurance measures and design modifications by endoscope manufacturers (e.g., duodenoscopes with a disposable tip cap to facilitate manual cleaning of the Albarran lever). With increasing prevalence of multidrug-resistant organisms (MDRO), coupled with improved capabilities in molecular genetic diagnostics, typing and the identification of chains of infection, outbreaks caused by MDRO following endoscopy are more easily detected and better traced. Several outbreaks caused by flexible gastrointestinal endoscopes involving multi-resistant *Klebsiella pneumoniae* [14], [15], [16] and *Pseudomonas aeruginosa* clones have been reported [17], [18], [19]. In many cases, inadequate reprocessing of endoscopes was identified as the cause. Provided that endoscopes are processed correctly and faulty endoscopes are taken out of service immediately (particularly in the case of defects in the working channel), it can be assumed that an adequate standard of reprocessing quality for reusable endoscopes will be achieved. If there are signs of wear and tear (e.g., scuff marks or other surface damage), particularly in the working channel of the endoscopes, a systematic inspection should be carried out. Outside of outbreak situations, the risk of duodenoscope-associated MDRO colonization or infection appears to be low, provided there is regular microbiological monitoring and strict quarantine protocols for endoscopes [20]. It should be noted, however, that despite all the advances in molecular genetic diagnostics, it can be difficult in clinical practice to detect and confirm pathogen transmission via endoscopes – or even endoscope-associated outbreaks – at an early stage, particularly if the causative pathogens do not exhibit any specific identifying characteristics (e.g., carbapenemase production). Especially as most of the pathogens in question are part of the normal human gastrointestinal microbiota (‘gut flora’), infections may occur with a time lag and in other settings, and many endoscopies are carried out on an outpatient basis. Negative results from microbiological sampling do not completely rule out subsequent contamination and may therefore merely represent a snapshot in time.

When evaluating the literature, it should be borne in mind that both reprocessing guidelines and the monitoring of reprocessing quality vary considerably from country to country. For example, the rigorous microbiological monitoring of both endoscopes and endoscope cleaning and washers/disinfectors (WD), which has been mandatory in Germany since 2002, is not currently recommended in other countries for every type of endoscope, nor is it carried out using sampling methods of comparable sensitivity.

**Until a valid comparison of endoscopes reprocessed in accordance with different specifications or guidelines is available, using a sample collection method of comparable sensitivity (e.g., the flush-brush-flush technique** **[7]** **), it is not possible to make a definitive assessment of the risk of endoscope-associated infection in relation to reprocessing methods and quality.**

## 3 Explanatory notes on the use of reusable versus single-use endoscopes

In general, it is difficult to draw sweeping conclusions about whether reusable or single-use medical devices are preferable, given the wide variety of products and the resulting diversity of requirements, for example, in terms of manufacture and reprocessing [21]. However, when comparing reusable and single-use endoscopes, sustainability aspects must also be taken into account alongside the assessment of the risk of endoscopy-associated infections, in particular the volume of waste generated and the consumption of chemicals, raw materials, water, and energy for manufacture and reprocessing.

As part of a cross-sectional study, the average volume of single-use waste generated by all endoscopies performed over a five-day period at two academic medical centers in the USA – one with a low volume of examinations and one with a high volume (2,000 and 13,000 endoscopic examinations per year, respectively) and the impact of switching from reusable to single-use endoscopes was calculated, taking into account the waste generated during reprocessing [22]. Each endoscopy generated 2.1 kg of single-use waste (46 l in volume). 64% of the waste was sent to landfill, 28% was biologically hazardous waste, and 9% was recycled. The estimated total waste generated annually from all endoscopic procedures in the USA is 38,000 tons (equivalent to the weight of 25,000 cars) and cover approximately 117 football pitches to a depth of one meter [22]. If all endoscopic procedures were carried out using single-use endoscopes instead of reusable endoscopes that had been properly reprocessed, the net volume of waste would increase by approximately 40%. Excluding waste from consumables, the net volume of waste arising from the reprocessing and disposal of endoscopes would quadruple if only single-use endoscopes were used [22]. Other factors that influence the life-cycle assessment, such as the consumption of raw materials, water or energy, were not directly taken into account here. A prospective, multicenter observational study showed that the extrapolated volume of waste generated by all endoscopic examinations in Germany is equivalent to that of a small town in Germany (population approx. 5,000–20,000) each year [23].

### 3.1 Impact on life-cycle assessment

#### General considerations

Currently, there are only a few analyses that compare the environmental impacts of reusable versus single-use endoscopes. The difficulty lies in the fact that environmental impacts are hard to quantify because reprocessing procedures – including the process chemicals used –vary by country, and the limited data available was collected under heterogeneous conditions. As a result of a theoretical case study, it was calculated that, excluding water consumption, reusable flexible bronchoscopes have a lower environmental impact in terms of global warming, abiotic resource depletion, ozone depletion, human toxicity, freshwater ecotoxicity, marine ecotoxicity, terrestrial ecotoxicity, photochemical oxidation, acidification, and eutrophication [24]. A prospective observational study found that single-use flexible bronchoscopes generated nearly twice as much recyclable waste as reusable devices. Only 15.8% of the waste generated by both device categories was recyclable [25]. A Scandinavian study, which employed a simplified method of life-cycle analysis based on the parameters of energy consumption, CO_2_ equivalent (eq) emissions and resource consumption, leaves open the question of which type of bronchoscope has the greatest impact on the environmental factors examined, as the examination and reprocessing procedures used, including the use of personal protective equipment (PPE), vary widely [26]. Unambiguous conclusions can only be drawn once life-cycle assessments from various countries are available that follow a standardized method [24].

#### Endoscope-specific considerations

Comparative data on reusable and single-use gastroscopes and duodenoscopes show that the manufacturing process for single-use devices has the greatest environmental impact [27], [28]. Le et al. concluded that, for single-use duodenoscopes, 91–96% of all greenhouse gas emissions are attributable to the production process [27]. The authors estimated that performing an ERCP with a single-use duodenoscope results in a CO_2_ eq of between 36.3 and 71.5 kg, which corresponds to a 24- to 47-fold higher CO_2_ emission than when using reusable duodenoscopes. Davis et al. found comparable environmental impacts associated with the use of flexible, reusable, and single-use ureteroscopes [29]. 

To assess the reprocessing procedure in terms of its impact on the life-cycle assessment, factors such as the consumption of process chemicals, water, and electricity must be considered. It is difficult to apply these findings to other contexts, as, among other things, the applicable regulatory frameworks for the reprocessing procedure must be taken into account in each case. Pioche et al. [28] investigated the environmental impact of a single-use gastroscope compared to a reusable gastroscope per examination. Reusable gastroscopes had a greater impact on the CO_2_ footprint when considering reprocessing: in terms of reprocessing, it generated 2.1 kg CO_2_-eq per examination and consumed 43.6 megajoules of fossil resources. In addition, the authors calculated that, from a life-cycle assessment perspective, the use of single-use gastroscopes may be justified for facilities performing fewer than 213 gastroscopies per year [28].

According to a U.S. study, assuming equivalent clinical performance, the benefits of switching from reusable bronchoscopes to single-use bronchoscopes depend largely on the hospital’s annual procedure volume, the expected risk of cross-infection and the purchase price of the bronchoscopes [30]. In a recent German publication by a university-affiliated tertiary care hospital, the cost per bronchoscope use was estimated at € 346.19 for single-use endoscopes and € 97.28 for reusable bronchoscopes. The break-even point was 317 uses per year. Sensitivity analyses demonstrated the robustness of the results in the face of rising maintenance costs as well as falling acquisition costs for single-use bronchoscopes [31]. Regardless of the number of endoscopies required for the use of single-use bronchoscopes to be cost-effective, it remains unclear whether their use is also more environmentally sustainable compared to reusable endoscopes. A systematic review evaluating flexible single-use cystoscopes found no differences in the length of hospital stay for patients examined with them or in complication rates. However, it did confirm a reduction in the working time of endoscopy staff. Cost-effectiveness and environmental impact depend largely on the number of cases and the available reprocessing options [32].

#### Consensus-building

An international Delphi consensus study, in which two representatives from each of 33 countries participated, evaluated the prerequisites for the use of single-use endoscopes [33]. Since Germany did not participate in this consensus-building process with its own representatives, the expert assessments contained therein – which were not collected in a representative manner – cannot be directly applied to the German healthcare system. According to the consensus study, the use of single-use endoscopes is generally recommended only if a well-functioning recycling system is in place locally (consensus: 94.6%) [33]. In principle, recycling of raw materials from single-use medical devices is possible at the point of waste generation [34]. However, in Germany, the national guidelines for the disposal of waste from healthcare facilities issued by the Federal/State Working Group on Waste (LAGA) must be taken into account, as they make material recycling significantly more difficult [35]. Individual pilot projects are increasingly exploring possible courses of action [36]. Data from Germany, collected specifically for endoscopy, show that even the proper recycling of uncontaminated packaging materials can help to reduce the volume of waste requiring disposal in accordance with the LAGA regulations. This directly reduces greenhouse gas emissions without compromising workflows or the safety of patients and staff [37]. 

A recent overview urges endoscopy facilities to take sustainability considerations into account (*“Reduce, reuse, recycle!”*) and emphatically concludes that the greatest savings in the life-cycle assessment can be achieved by avoiding endoscopies that are not medically indicated [38]. Another factor that should not be underestimated when calculating the life-cycle assessment of endoscopy units is the transportation of staff and patients to the examination site [23]. In cases where endoscopies are indicated, combining procedures (e.g., gastroscopy and colonoscopy) can save resources.

#### Manufacturer initiatives

Efforts by various manufacturers to reduce their environmental impact include reducing product weight, minimizing packaging, using recyclable secondary packaging, providing a reusable power cord, and shortening the distance between the production facility and the factory [24].

## 4 Technical quality of reusable versus single-use endoscopes

In Germany, according to Section 23 of the Infection Protection Act in Germany (IfSG), the KRINKO has the official mandate to develop national recommendations for the prevention of healthcare-associated infections in healthcare and nursing facilities [39]. Statements regarding the technical aspects of medical procedures (e.g., consideration of diagnostic benefits) are not usually addressed but should be taken into account when deciding whether to use a reusable or single-use endoscope. Determining device preferences solely based on technical parameters is not productive, as many situation-specific factors play a role in the assessment (e.g., clinical application, endoscope types, endoscopy experience of the examiner and assistant, patient’s anatomy).

According to the participants in the above-mentioned consensus study, single-use endoscopes should deliver diagnostic results comparable to those of reusable endoscopes (consensus 96.5%) [33]. This can be illustrated, for example, by a small randomised controlled trial of duodenoscopies involving therapeutic ERCP, which directly compared reusable (n=50) versus single-use duodenoscopes (n=48). The primary endpoint was the number of attempts required to successfully cannulate the bile duct and/or pancreatic duct. In the group using single-use duodenoscopes, the median number of successful cannulation attempts was significantly lower (p=0.013). Furthermore, reusable duodenoscopes performed significantly better in several technical aspects, e.g., with regard to gastric passage, image quality, image stability and the functionality of the air-water valve button [40]. 

## 5 Practical considerations regarding the use of reusable and single-use endoscopes from an infection prevention perspective

**Current practice involves the use of reusable endoscopes. The use of single-use endoscopes may be considered.**

By carefully considering the situations in which reusable or single-use endoscopes should be used, on a facility-by-facility basis, it is possible to reduce resource consumption and environmental impact.

**General considerations as to the situations in which the use of single-use endoscopes might be appropriate from an infection-prevention perspective include (examples):**

- Suspected (or confirmed) Creutzfeldt-Jakob disease/variant Creutzfeldt-Jakob disease (CJD/vCJD), as the standard reprocessing procedure at WD does not sufficiently guarantee the decontamination of prions. The reprocessing of reusable endoscopes is only possible at centers with a specifically validated reprocessing protocol for CJD [7].
- Suspected (or confirmed) high-consequence infectious disease, in particular viral haemorrhagic fevers (e.g., Ebola, Lassa fever, Crimean-Congo haemorrhagic fever), with the use of single-use endoscopes making the procedure considerably easier.
- Situations in which it is not possible to reprocess the equipment immediately, e.g., during endoscopy on weekend duty, particularly in smaller hospitals.
- Outbreak situations in which it is suspected that reusable endoscopes are the cause, due to shortcomings in the reprocessing procedure, or are playing a role in the outbreak.
- Very infrequent use of specific types of endoscope, which makes reprocessing at WD economically challenging.

The findings of the Delphi consensus study mentioned above cannot be directly applied due to the high standards of data processing in Germany; however, they are intended to highlight the risk assessment criteria that need to be taken into account [33]. For example, the consensus study recommends the use of single-use endoscopes in cases of known colonization or infection with MDRO (consensus 94.8%) [33]. However, this cannot be justified on the basis of robust data, as the microbial colonization of patients is generally unknown. Consequently, reprocessing must be carried out in accordance with quality assurance standards, regardless of specific risk scenarios, with regular process and product controls using suitable, sufficiently sensitive methods [7]. Pre-procedural MDRO screening of patients undergoing duodenoscopy with ERCP also showed that the overall quality of endoscope reprocessing is a key factor in successfully preventing endoscope-associated contamination and infections, even in settings with a high prevalence of MDRO [41]. These data emphasize that single-use endoscopes are not required for the management of patients with MDRO colonization or infection. 

## 6 Conclusion

Reusable endoscopes may, despite the greater effort required for their reprocessing, be more environmentally beneficial overall than single-use products, particularly where they are used frequently and reprocessing procedures are optimized. Single-use endoscopes, on the other hand, generate more waste and consume more resources per use, but may offer advantages in certain situations in terms of infection prevention and logistical simplicity. It is therefore crucial to weigh up environmental considerations, patient safety, practical feasibility and economic factors in a nuanced manner. Overall, there are strong arguments in favor of the targeted, indication-based use of both systems. The internal decisions regarding the use of reusable versus single-use endoscopes require a precise understanding of the patients to be treated, the scope of care, the site-specific care structures and the procedure-specific risks.

## Notes

The German original of this narrative review was published in the Epidemiologisches Bulletin: 

Lübbert C, Mutters NT, Scheithauer S, Baum H von, Meining A, Rieß A et al. Einsatz von Mehrweg- bzw. Einwegendoskopen aus infektionspräventiver Sicht unter besonderer Berücksichtigung der Nachhaltigkeit. Epidemiologisches Bulletin 2026; (33):3-12. DOI: 10.25646/14304

### Authors’ ORCIDs

- Lübbert C: https://orcid.org/0000-0001-6942-5785
- Mutters NT: https://orcid.org/0000-0002-0156-9595
- Scheithauer S: https://orcid.org/0000-0003-0773-4739
- Thanheiser M: https://orcid.org/0009-0005-4139-6544
- Lexow F: https://orcid.org/0000-0003-3463-2842
- Maidhof J: https://orcid.org/0009-0000-3884-8347
- Meining A: https://orcid.org/0009-0001-3773-138X
- Kramer A: https://orcid.org/0000-0003-4193-2149

### Funding

None. 

### Competing interests

The authors declare that they have no competing interests.

Prof. Dr. Christoph Lübbert, Prof. Dr. Nico T. Mutters, Prof. Dr. Simone Scheithauer, Prof. Dr. Heike von Baum, Andreas Rieß, and Prof. em. Dr. Axel Kramer are members of the Commission for Infection Prevention and Hygiene in Healthcare and Nursing (KRINKO). However, this publication does not represent an official recommendation or statement of the commission.

### AI usage statement 

No artificial intelligence (AI) tools were used in the conception, design, data collection, data analysis, interpretation of the results, or preparation of this manuscript except the use of DeepL for the rough translation. All aspects of the work were carried out solely by the authors.
